# Computational study of the motor neuron protein KIF5A to identify nsSNPs, bioactive compounds, and its key regulators

**DOI:** 10.3389/fgene.2023.1282234

**Published:** 2023-11-10

**Authors:** Rupesh Kumar, Thirumurthy Madhavan, Kalaiarasan Ponnusamy, Honglae Sohn, Shazia Haider

**Affiliations:** ^1^ Department of Biotechnology, Jaypee Institute of Information Technology, Noida, Uttar Pradesh, India; ^2^ Department of Genetic Engineering, Computational Biology Lab, SRM Institute of Science and Technology, Chennai, India; ^3^ Biotechnology Divison, National Centre for Disease Control, New Delhi, India; ^4^ Department of Chemistry and Department of Carbon Materials, Chosun University, Gwangju, Republic of Korea; ^5^ Department of Biosciences, Jamia Millia University, New Delhi, India

**Keywords:** neurodegenerative diseases, amyotrophic lateral sclerosis, kinesin family member 5A, nonsynonymous single-nucleotide polymorphisms, protein‐protein interaction, phytochemicals, miRNA, transcription factor

## Abstract

**Introduction:** Kinesin family member 5A (*KIF5A*) is a motor neuron protein expressed in neurons and involved in anterograde transportation of organelles, proteins, and RNA. Variations in the KIF5A gene that interfere with axonal transport have emerged as a distinguishing feature in several neurodegenerative disorders, including hereditary spastic paraplegia (HSP10), Charcot-Marie-Tooth disease type 2 (CMT2), and Amyotrophic Lateral Sclerosis (ALS).

**Methods:** In this study, we implemented a computational structural and systems biology approach to uncover the role of KIF5A in ALS. Using the computational structural biology method, we explored the role of non-synonymous Single Nucleotide Polymorphism (nsSNPs) in *KIF5A*. Further, to identify the potential inhibitory molecule against the highly destabilizing structure variant, we docked 24 plant-derived phytochemicals involved in ALS.

**Results:** We found KIF5A^S291F^ variant showed the most structure destabilizing behavior and the phytocompound “epigallocatechin gallate” showed the highest binding affinity (−9.0 Kcal/mol) as compared to wild *KIF5A* (−8.4 Kcal/mol). Further, with the systems biology approach, we constructed the *KIF5A* protein-protein interaction (PPI) network to identify the associated Kinesin Families (KIFs) proteins, modules, and their function. We also constructed a transcriptional and post-transcriptional regulatory network of *KIF5A*. With the network topological parameters of PPIN (Degree, Bottleneck, Closeness, and MNC) using CytoHubba and computational knock-out experiment using Network Analyzer, we found KIF1A, 5B, and 5C were the significant proteins. The functional modules were highly enriched with microtubule motor activity, chemical synaptic transmission in neurons, GTP binding, and GABA receptor activity. In regulatory network analysis, we found *KIF5A* post-transcriptionally down-regulated by miR-107 which is further transcriptionally up-regulated by four TFs (HIF1A, PPARA, SREBF1, and TP53) and down-regulated by three TFs (ZEB1, ZEB2, and LIN28A).

**Discussion:** We concluded our study by finding a crucial variant of KIF5A and its potential therapeutic target (epigallocatechin gallate) and *KIF5A* associated significant genes with important regulators which could decrypt the novel therapeutics in ALS and other neurodegenerative diseases.

## Introduction

Kinesin molecular motor proteins are ATPase-dependent machinery that move along microtubule tracks to promote cargo transport within the cell. Motor proteins mainly consist of binding sites for cargo transport and interaction with tubulin subunits. Since its discovery, many members of the kinesin family (KIF) have been identified (Nakagawa et al., 1997; Miki et al., 2001), which is divided into 15 kinesin families, referred to as kinesin 1 to kinesin 14B (Hirokawa et al., 2009; Wang and Xu, 2015). Functional impairment, imaging techniques, and biochemical investigations have all revealed significant functions of kinesins in the process of cell division (Sekine et al., 1994; He et al., 2016), brain development (Hirokawa et al., 2010), and many neurological disorders such as Alzheimer’s disease (AD) (Kamal et al., 2001), amyotrophic lateral sclerosis (ALS) (Devon et al., 2006; Had et al., 2006), and hereditary spastic paraplegia (HSP10) (Reid et al., 2002). Multiple studies have indicated that kinesins play specific and crucial roles in synaptic transmission (Hirokawa et al., 2009; Chen et al., 2011; Kunz et al., 2013). Nonetheless, it remains uncertain whether only specific subsets or all members of kinesins are necessary for synaptic transmission, and the mechanisms through which KIFs govern excitatory synaptic transmission are yet to be fully understood.

*KIF5A* is a member of kinesin superfamily proteins. It is expressed in neuronal cells and plays an important role in axonal transport (Smith et al., 2006). *KIF5A* consists of a motor domain at N-terminal, a central coiled-coil stalk region, and a C-terminal cargo binding region (Smith et al., 2006). Studies have elucidated that mutation in N-terminal domains and cargo binding at the C-terminal region of *KIF5A* cause aggregation of abnormal proteins, metabolic dysfunction, cell-to-cell communication dysregulation, and alternation in genetic networks, leading to escalation of neuronal cell death (Valko and Ciesla, 2019). Interestingly, missense mutation in the N-terminal motor domain and the central stalk domain of *KIF5A* is known to be related to autosomal diseases including hereditary spastic paraplegia (Reid et al., 2002; Goizet et al., 2009; Morais et al., 2017) and Charcot–Marie–Tooth type 2 (CMT2) (Liu et al., 2014). Additionally, it has been found that splice site mutations in the C-terminal binding domain of *KIF5A* cause exon skipping, leading to the loss of RNA expression. Thus, it is suggested that haploinsufficiency is the most likely underlying molecular mechanism for the neurodegenerative disorder ALS (Brenner et al., 2018). It is the foremost neurodegenerative disease that arises in two forms, familial and sporadic ALS majorly persistent motor neuron disease, with an occurrence of 1–5 for every 100,000 individuals (Hardiman et al., 2017). Because of the absence of compelling treatment, ALS prompts death between 2 and 5 years after diagnosis, majorly because of the failure of respiratory tract function. However, most of the cases are sporadic (sALS) (Van Rhe et al., 2016), but only 5%–10% are associated with a genetic mutation that is inherited through the family (Chia et al., 2018). One of the recent studies has revealed that a non-synonymous SNP variant (rs113247976) in *KIF5A*, located in the C-terminal region [Pro986Leu] of the exon, plays a significant role in ALS (Brenner et al., 2018). Impairments in axonal transport and modifications in the cytoskeletal structure are prominent characteristics observed in ALS patients, primarily contributing to the degeneration of motor neuron pathways (Roy et al., 2010). Missense mutations within the C-terminal domain have been notably linked to disruptions in microtubule binding and ATP hydrolysis, resulting in the impairment of anterograde transport of cargo proteins within both dendritic and axonal regions (Ebbing et al., 2008). The primary cause of ALS is the loss of function resulting from a missense mutation in the C-terminal domain of KIF5A. Thus, there is an urgent need for preventive measures that can effectively restore or stabilize the loss of cytoskeleton function caused by mutations. This provides an opportunity for the development of drugs that could potentially address ALS. There are several non-phytochemical-based therapies used to treat neurodegenerative diseases and amyotrophic lateral sclerosis, and some of them had faced challenges or failures in clinical trials. Here, we present some current therapies and examples of drugs with their mechanisms of action and known challenges or failures: monoclonal antibodies targeting amyloid beta or tau (e.g., aducanumab for Alzheimer’s disease) aim to clear amyloid-beta plaques or tau aggregates (Zhang et al., 2023). Aducanumab’s FDA approval in 2021 was controversial and failed due to reasons of clinical efficacy and high cost. Glutamate modulators (e.g., memantine for Alzheimer’s disease) are NMDA receptor antagonists that regulate glutamate activity to protect neurons from excitotoxicity (Wang and Reddy, 2017). Memantine is used in Alzheimer’s treatment but may provide only modest benefits in some cases. Gene therapy (e.g., onasemnogene abeparvovec for spinal muscular atrophy) used to deliver a functional copy of the *SMN1* gene to replace the defective gene in spinal muscular atrophy (SMA) has shown clinical benefits (Ogbonmide et al., 2023). Riluzole modulates glutamate activity and may slow the progression of ALS by reducing excitotoxicity (Jiang et al., 2022). Riluzole is an approved drug for ALS treatment and can extend survival by several months. Edaravone (Radicava) is an antioxidant that aims to reduce oxidative stress, which may contribute to motor neuron damage in ALS. While edaravone received FDA approval for ALS treatment, its clinical benefits are modest and primarily seen in specific patient subgroups (Jiang et al., 2022). Both riluzole and edaravone do not provide a permanent treatment to ALS patients (Jaiswal, 2019). Another antisense oligonucleotide therapy tofersen (formerly IONIS-SOD1Rx) to target SOD1 gene expression has been formulated. The drug showed promise in early-stage clinical trials, and further trials were underway to assess its efficacy (Miller et al., 2022). It is essential to recognize that neurodegenerative diseases and ALS are complex, heterogeneous conditions with multiple underlying mechanisms, and finding effective treatments remains a significant challenge. While some therapies have been successful in managing symptoms or slowing disease progression to some extent, there is still a need for more targeted and curative approaches. Ongoing research efforts continue to explore novel treatments and mechanisms to address these devastating diseases.

Here, we applied structural and systems biology approaches to identify novel molecules for drug design against ALS. Genetic variations play a crucial role in the process of evolution, as they are linked to the phenotypic effects that can shed light on various disorders (Venselaar et al., 2010). SNPs can damage the protein structure, stability, function, and activity (Wu and Jiang, 2013). Our study focused on identifying the plant-derived bioactive molecules involved in excitatory amino acid toxicity, neuroinflammation, calcium cytotoxicity, and oxidative stress (Mohi-ud-din et al., 2021) in ALS against highly damaging missense SNPs (Yue and Moult, 2006). The phytochemicals from plant sources, such as flavonoids, alkaloids, terpenes, and saponins, may still have the potential benefits that researchers are seeking, which is because of their distinctive chemical diversity (Solanki et al., 2015; Zeng et al., 2021; Shareena and Kumar). Some of these phytochemicals cannot be synthesized by currently known methods. Therefore, these natural substances are still unexplored as new therapeutic molecules for the treatment of ALS (Matheus et al., 2023). The systems biology approaches have played an important role in identifying novel disease network regulators in neurological diseases (Su et al., 2018). We constructed and analyzed the protein–protein interaction (PPI) network of *KIF5A* to identify the subnetworks involved in a particular function and identification of other KIF members that interacted with *KIF5A* using network analysis. Furthermore, we performed the GO enrichment analysis of subnetworks in terms of biological processes (BPs), molecular functions (MFs), and cellular components (CCs). Transcriptional and post-transcriptional regulation is an important mechanism for the cell to have a normal function. Notably, one gene may be regulated by multiple miRNAs and transcription factors (TFs), and each miRNA and TFs target more than one gene. The regulatory molecule transcription factors (transcription regulators: TFs) and microRNA (post-transcriptional regulators: miRNA) help elucidate the expression level of genes (Su et al., 2018). Computational predicted miRNA targets and transcription factors of *KIF5A* were extracted from databases to understand the regulatory mechanisms.

## Methods

### Data mining for SNPs

**FIGURE 1:**
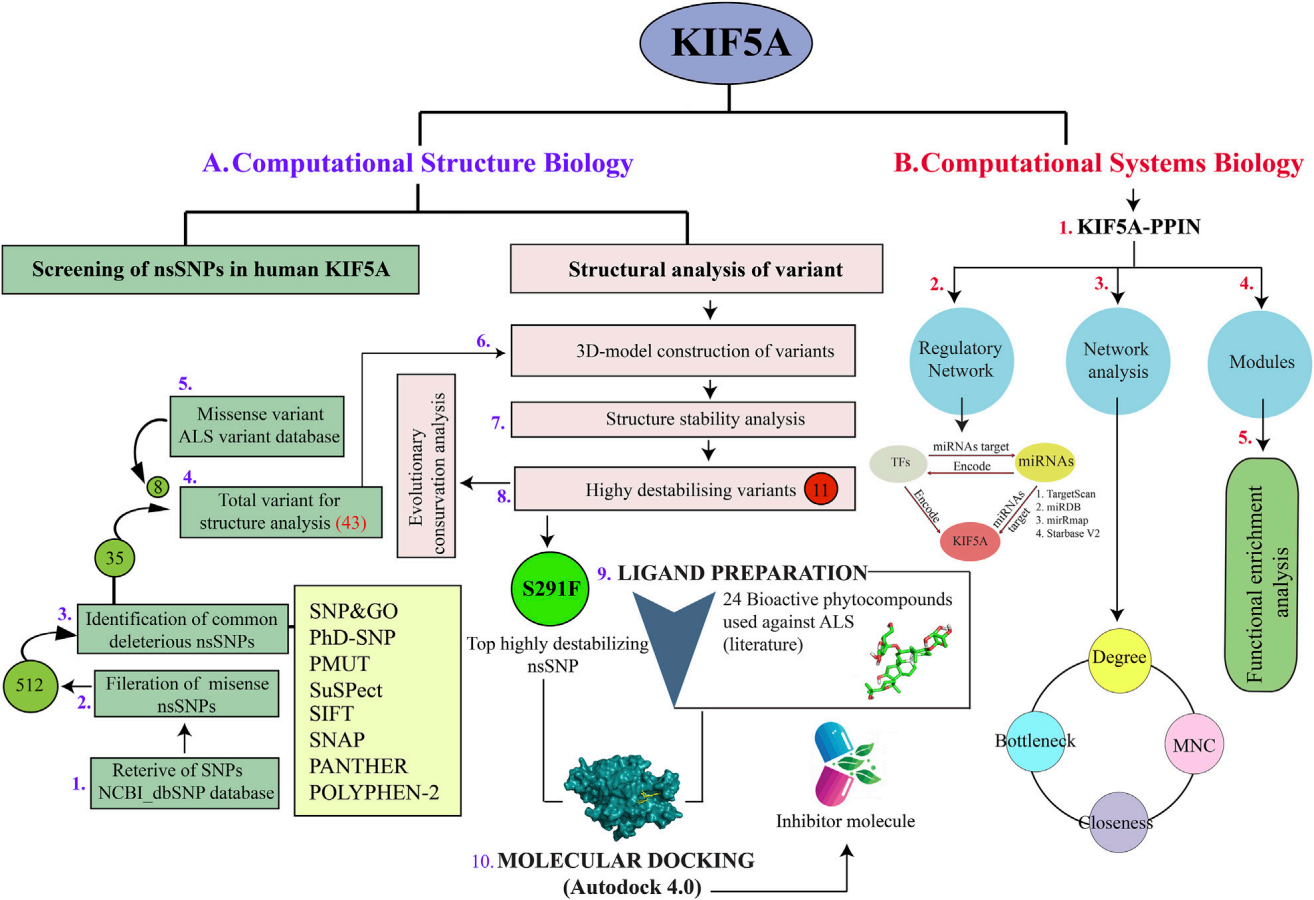
Implemented workflow used during the structure and systems biology approach in the *KIF5A* motor neuron protein. **(A)** Retrieval of SNPs using the dbSNP database used to filter missense SNPs (512), which was further used for identifying common deleterious nsSNPs (35) using eight different prediction tools. We also included eight nsSNPs associated with KIF5A, given in the ALS Variant database. Out of a total of 43 nsSNPs, we screened 11 destabilizing *KIF5A* variants, in which mutant S291F was the top destabilizing nsSNP. We prepared 24 bioactive compounds to screen the potential inhibitors of mutant S291F using molecular docking analysis. **(B)** Schematic analysis of PPIN of KIF5A included the regulatory network analysis using transcriptional and post-transcriptional molecules, transcription factors, and miRNAs. Identification of *KIF5A* associated functional modules; the KIF5A–PPIN network analysis to identify KIFs using the network parameters degree, bottleneck, MNC, and closeness.

Retrieval of protein sequences and SNPs of *KIF5A* has been carried out from the dbSNP database build 155 on NCBI (https://www.ncbi.nlm.nih.gov/gene/3798) and ALS Variant Server (http://als.umassmed.edu/). Here, our search majorly focused on non-synonymous missense SNPs, which were subsequently subjected to analysis to determine their detrimental and disease-causing impacts on the *KIF5A* protein; the detailed schematic representation of the study is provided in Figure 1A. We used eight different sequences and structure-based tools SIFT (Ng and Henikoff, 2003), PolyPhen-2 (Adzhubei et al., 2013), SNAP^2^ (Bromberg and Rost, 2007), PhD-SNP (Capriotti and Fariselli, 2017), PMut (López-Ferrando et al., 2017), SNPs&GO (Capriotti et al., 2013), PANTHER (Tang and Thomas, 2016), and SuSPect (Yate et al., 2014) to analyze the functional effects of nsSNPs. Alongside common nsSNPs screened using eight different prediction programs, we incorporated an additional eight nsSNPs reported in the ALS Variant Database (http://als.umassmed.edu/). These eight nsSNPs were not presented in the NCBI dbSNP database build 155 during the screening of nsSNPs. Consequently, to comprehensively assess their structural impact and their influence on protein stability, we included them in our analysis (Figure 1A) (Nicolas et al., 2018; Bertolin et al., 2017).

### 3D model construction and validation

The three-dimensional structure of *KIF5A* was downloaded from UniProt (https://www.uniprot.org/uniprotkb/Q12840/entry). The wild-type structure of *KIF5A* was evaluated in PDBsum (http://www.ebi.ac.uk/thornton-srv/databases/pdbsum/Generate.html) that provides a visual representation of molecules, including the construction of structures like DNA, protein chains, ligands, and metal ions, along with a schematic depiction of their interactions (Yang et al., 2014). The Ramachandran plot assesses the dihedral angles of amino acid residues, and by analyzing their phi and psi dihedral angles, it identifies which residues are energetically permissible. This process helps establish the structural and functional characteristics of a protein (Gopalakrishnan et al., 2007; Ca et al., 2014). PDBsum offers a visual representation of a protein’s three-dimensional structure and provides information on angles, helices, motifs, beta-sheets, and strands. An ideal protein structure typically comprises over 90% of its residues within the favored region (Laskowski et al., 2018). PROCHECK evaluates the stereochemical characteristics of proteins by examining both the overall structural and residue-level geometry (https://www.ebi.ac.uk/thornton-srv/software/PROCHECK/) (Laskowski et al., 1993).

### Stability change prediction of mutants

The structure of the mutants was generated using the WHAT IF server (https://swift.cmbi.umcn.nl/servers/html/index.html) (Vriend, 1990), and further energy minimization of mutants and native structure was performed using YASARA (http://www.yasara.org/minimizationserver.htm) (Krieger et al., 2009). The structural stability of the mutant for the wild-type was predicted utilizing FoldX (http://foldxsuite.crg.eu/) (Schymkowitz et al., 2005). It generates a quantitative estimation of significant interactions imparting to the stability of protein structure by utilizing its atomic description (Schymkowitz et al., 2005).

### Domain prediction, evolutionary conservation, and mapping of highly deleterious nsSNPs

To predict the functional domain of *KIF5A*, we used the InterPro server (https://www.ebi.ac.uk/interpro/), version 95.0, accessed on 8 July 2023. InterPro provides the functional analysis of proteins by classifying them into families and predicting domains and important sites (Hunter et al., 2009). InterPro combines protein signatures from 13 member databases CATH-Gene3D, CDD, HAMAP, PANTHER, Pfam, PIRSF, PRINTS, PROSITE profiles, PROSITE patterns, SFLD, SMART, SUPERFAMILY, and TIGRFAMs (Hunter et al., 2009). The ConSurf web server was employed to compute the evolutionary conservation of amino acid positions (Ashkenazy et al., 2016) by assigning of a score between 1 (most variable position) and 9 (most conserved position) to each amino acid position. The protein sequence similarity searching was performed against UniRef90, in which CSI-BLAST (Context-Specific Iterated-Basic Local Alignment Search Tool), 3, and 0.0001 were set for the homolog search algorithm, number of iterations, and E-value cutoff, respectively. Furthermore, we mapped the predicted highly structure destabilizing nsSNPs against the functional domain of *KIF5A* to understand their functional impact (Figure 1A).

### Selection of bioactive plant-derived compounds and molecular docking

Bioactive compounds derived from plants are being explored for their potential effectiveness against excitatory amino acid toxicity, neuroinflammation, calcium cytotoxicity, and oxidative stress in ALS as observed from the literature (Mohi-ud-din et al., 2021). These compounds were downloaded from ZINC and PubChem (https://pubchem.ncbi.nlm.nih.gov/databases) and were further pre-processed using AutoDock 4.0 (Eberhardt et al., 2021). To understand the mechanism of action of plant-derived bioactive compounds showing the highest binding affinity with the KIF5A^S291F^ mutant, we performed a comparative docking study in the wild-type *KIF5A* kinesin motor domain and the mutant KIF5A^S291F^ structure to calculate their binding affinity and interacting residues.

### *KIF5A–*PPIN and its functional modules

A network theory-based approach is used to delineate connections within intricate systems and serves as an important organizing principle for cellular networks within the field of systems biology. This approach further facilitates the comprehension of how specific molecules contribute to various cellular processes. We constructed the PPIN of *KIF5A* using the STRING database (Szklarczyk et al., 2019) and identified the functional modules using “Molecular Complex Detection” (MCODE) v2.0.0 (Hogue and Groll, 2001). MCODE is a Cytoscape plugin that is designed to pinpoint nodes with high interconnections forming clusters that represent relatively stable, multi-protein complexes functioning as unified entities within the network. Modules control the network from dissortivity and also maintain the flow of information throughout the network (Lalwani et al., 2022; Nafis et al., 2014). The identified modules were functionally annotated in terms of BPs, MFs, and CCs using the Enrichr web-based tool to correlate the biological significance (Xie et al., 2021). We further analyze the PPIN of *KIF5A* using two local ranking methods degree (Pk) and maximum neighborhood component (MNC) and two global methods (bottleneck and closeness) with the help of the Cytoscape (v3.8.0) (Shannon et al., 2003) plugin CytoHubba (Chin et al., 2014). Using CytoHubba, we can identify the top 10 nodes which could play an important role in controlling the network and signal propagation (Chen et al., 2023; Takayama et al., 2023; Gu et al., 2023). We also performed the computational knockout experiments by eliminating the top significant node, resulting in network perturbations, a phenomenon known as the centrality–lethality rule (Jeong et al., 2001; Malik et al., 2019). Accumulating mutations in a gene can lead to the disruption or loss of expression of a particular protein, resulting in its impairment or absence within the cell. In general, this situation of re-examining the network parameter can be illustrated by eliminating the nodes that may have been modified from the network (Peng et al., 2015). Here, we computed the topological parameters such as degree distribution (Pk), betweenness centrality (BC), and neighborhood connectivity (NC) of the modified/reorganized network to assess the regulating capacities of nodes using Network Analyzer in Cytoscape (v3.8.0) (Shannon et al., 2003).

### Tracing the key transcriptional and post-transcriptional regulators of KIF5A

To understand the post-transcriptional regulation of *KIF5A*, we identify the number of miRNA targets by using four different databases, TargetScanHuman release 8.0 (Agarwal et al., 2015), miRDB (Chen and Wang, 2020), miRmap (Vejnar and Zdobnov, 2012), and starBase v2.0 (Li et al., 2014). The TFs related to *KIF5A* were extracted using the ENCODE in-built option in NetworkAnalyst 3.0, a comprehensive gene expression profiling and network visual analytic tool (Zhou et al., 2019). To identify the key transcriptional and post-transcriptional regulators of *KIF5A*, we mapped the TFs and the common miRNA targets present in all four miRNA target prediction databases. We constructed and visualized the regulatory network of *KIF5A*–miRNAs–TFs using Cytoscape v3.8.0 (Shannon et al., 2003).

## Results

### Deleterious nsSNPs in *KIF5A*

The *in silico* screening of 511 nsSNPs was performed using sequence- and structure-based tools (Figure 1A). The eight screening programs SIFT, PolyPhen-2, SNAP^2^, PhD-SNP, PMut, SNPs&GO, PANTHER, and SuSPect identified 35 common nsSNPs as deleterious and could have a high chance of affecting the protein function (Table 1). We added eight more missense variants of exonic region SNPs of *KIF5A* (E669K, E758K, T858A, T947A, T887I, T976I, P897L, and P986L) retrieved from the ALS Variant Server to understand their deleterious impact on structure stability for further analysis.

**TABLE 1:** List of 35 common nsSNPs identified as deleterious in eight different prediction tools having a high chance of affecting the protein function (*KIF5A*).

dbSNP rs#	WposM	SuSPect pred	SuSPect score	PMut pred	PMut score	SNAP pred	SNAP score	PolyPhen pred	SIFT pred	SIFT score	M-score	SNPs&GO	RI	PANTHER	Preservation time	PhD-SNP	RI
rs770302674	P18S	D	75	D	0.7831	E	59	HD	APF	0	3.02	D	6	PD	1,628	D	8
rs1060502525	G77D	D	91	D	0.8555	E	83	HD	APF	0	3.02	D	7	PD	1,628	D	9
rs1299838179	N79S	D	83	D	0.8424	E	52	HD	APF	0	3.02	D	6	PD	1,628	D	8
rs1060502524	S90L	D	93	D	0.8264	E	53	HD	APF	0	3.02	D	5	PD	1,628	D	6
rs1237275454	M96V	D	89	D	0.8555	E	51	HD	APF	0	3.02	D	3	PD	1,628	D	2
rs978901860	R144C	D	66	D	0.5085	E	66	HD	APF	0.01	3.02	D	6	PD	1,237	D	6
rs1060502522	D145H	D	80	D	0.8555	E	79	HD	APF	0	3.02	D	6	PD	1,237	D	8
rs1469562271	E158K	D	66	D	0.8424	E	55	HD	APF	0	3.02	D	4	PD	1,368	D	6
rs1191334617	D159G	D	56	D	0.6906	E	55	HD	APF	0.02	3.02	D	3	PD	1,036	D	3
rs749561961	S176R	D	51	D	0.7455	E	48	HD	APF	0.01	3.02	D	3	PD	1,237	D	4
rs147510678	G187A	D	61	D	0.8424	E	54	HD	APF	0.01	3.02	D	4	PD	1,628	D	5
rs769315791	R191C	D	86	D	0.8555	E	94	HD	APF	0	3.02	D	6	PD	1,628	D	8
rs1488871976	R191H	D	82	D	0.8555	E	96	HD	APF	0	3.02	D	5	PD	1,628	D	7
rs773071687	A194V	D	71	D	0.8555	E	58	HD	APF	0	3.02	D	3	PD	1,628	D	1
rs1057524193	S202R	D	83	D	0.8424	E	76	HD	APF	0	3.02	D	4	PD	1,628	D	6
rs1057519195	S202T	D	79	D	0.8555	E	64	HD	APF	0	3.02	D	3	PD	1,628	D	3
rs387907287	R204Q	D	73	D	0.8555	E	86	HD	APF	0	3.02	D	5	PD	1,628	D	6
rs690016545	D232N	D	79	D	0.8555	E	90	HD	APF	0.01	3.02	D	3	PD	1,628	D	6
rs387907289	G235E	D	71	D	0.8555	E	80	HD	APF	0	3.02	D	6	PD	1,628	D	8
rs387907285	E251K	D	72	D	0.8555	E	75	HD	APF	0	3.02	D	6	PD	1,628	D	8
rs121434441	N256S	D	79	D	0.8555	E	53	HD	APF	0	3.02	D	6	PD	1,628	D	8
rs947028044	K257M	D	55	D	0.8316	E	54	HD	APF	0.01	3.02	D	1	PD	1,628	D	2
rs1174199852	V265A	D	80	D	0.8555	E	48	HD	APF	0	3.02	D	2	PD	1,628	D	4
rs121434443	Y276C	D	62	D	0.7914	E	62	HD	APF	0	3.02	D	5	PD	455	D	5
rs1291404967	V277F	D	84	D	0.8424	E	85	HD	APF	0	3.02	D	5	PD	456	D	7
rs121434442	R280C	D	80	D	0.8555	E	55	HD	APF	0	3.02	D	6	PD	1,628	D	7
rs387907288	R280H	D	73	D	0.8555	E	66	HD	APF	0	3.02	D	5	PD	1,628	D	6
rs1057523746	K283E	D	58	D	0.8555	E	80	HD	APF	0	3.02	D	6	PD	1,628	D	6
rs761812789	S291F	D	89	D	0.8555	E	76	HD	APF	0	3.02	D	5	PD	1,628	D	7
rs1416085161	R297W	D	65	D	0.8396	E	81	HD	APF	0	3.02	D	5	PD	1,628	D	5
rs1303126235	T298M	D	90	D	0.8424	E	67	HD	APF	0	3.02	D	3	PD	1,628	D	5
rs1290797018	M300T	D	86	D	0.8555	E	69	HD	APF	0	3.02	D	1	PD	455	D	6
rs1305787310	P306A	D	69	D	0.8357	E	41	HD	APF	0	3.02	D	2	PD	1,628	D	2
rs1012819766	R323W	D	81	D	0.8434	E	90	HD	APF	0	3.02	D	4	PD	1,628	D	7
rs372633156	R798C	D	53	D	0.5082	E	24	HD	APF	0	3.32	D	1	PD	1,036	D	6

Abbreviations: D (disease); E (effect); HD (highly damaging); APF (affect protein function); PD (probably damaging).

### 3D model of *KIF5A*, validation, and mutant stability comparison

**FIGURE 2:**
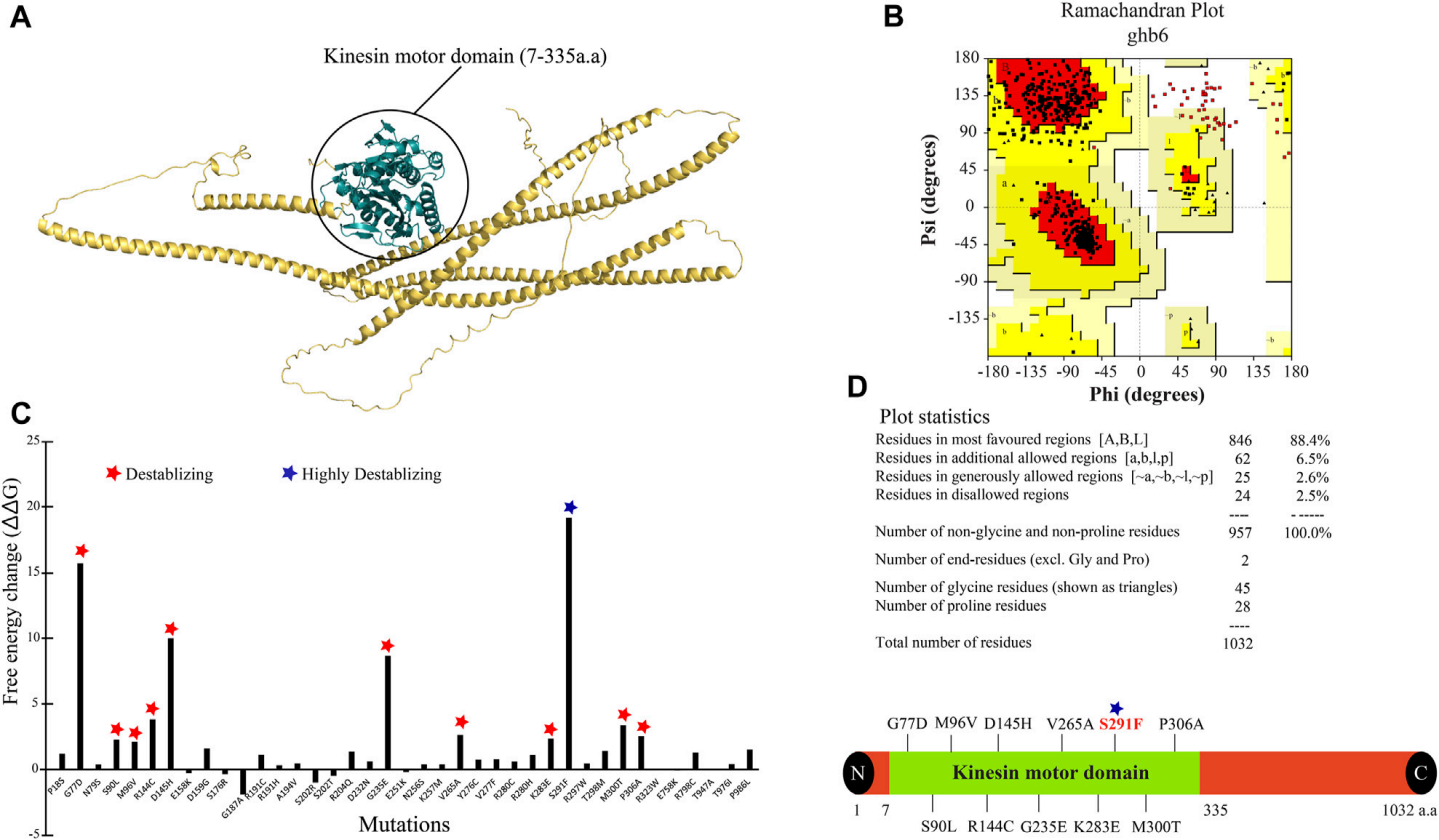
*KIF5A* protein structure, validation, mutant stability analysis, and their mapping. **(A)** The 3D structure of *KIF5A* protein having a sequence length of 1,032 showing the kinesin motor domain in deep teal color (7–335 amino acids). **(B)** The representation of the Ramachandran plot generated by PDBsum stated the information of amino acids in a 3D structure. **(C)** Highlighting the free energy differences between 43 mutants, in which “S291” showed the highest destabilizing nature. **(D)** Representation of destabilizing mutants in the kinesin motor domain; out of 12 destabilizing mutants, 11 were presented in the kinesin motor domain (7–335 amino acids), and the S291F mutant is shown in red text.

**FIGURE 3:**
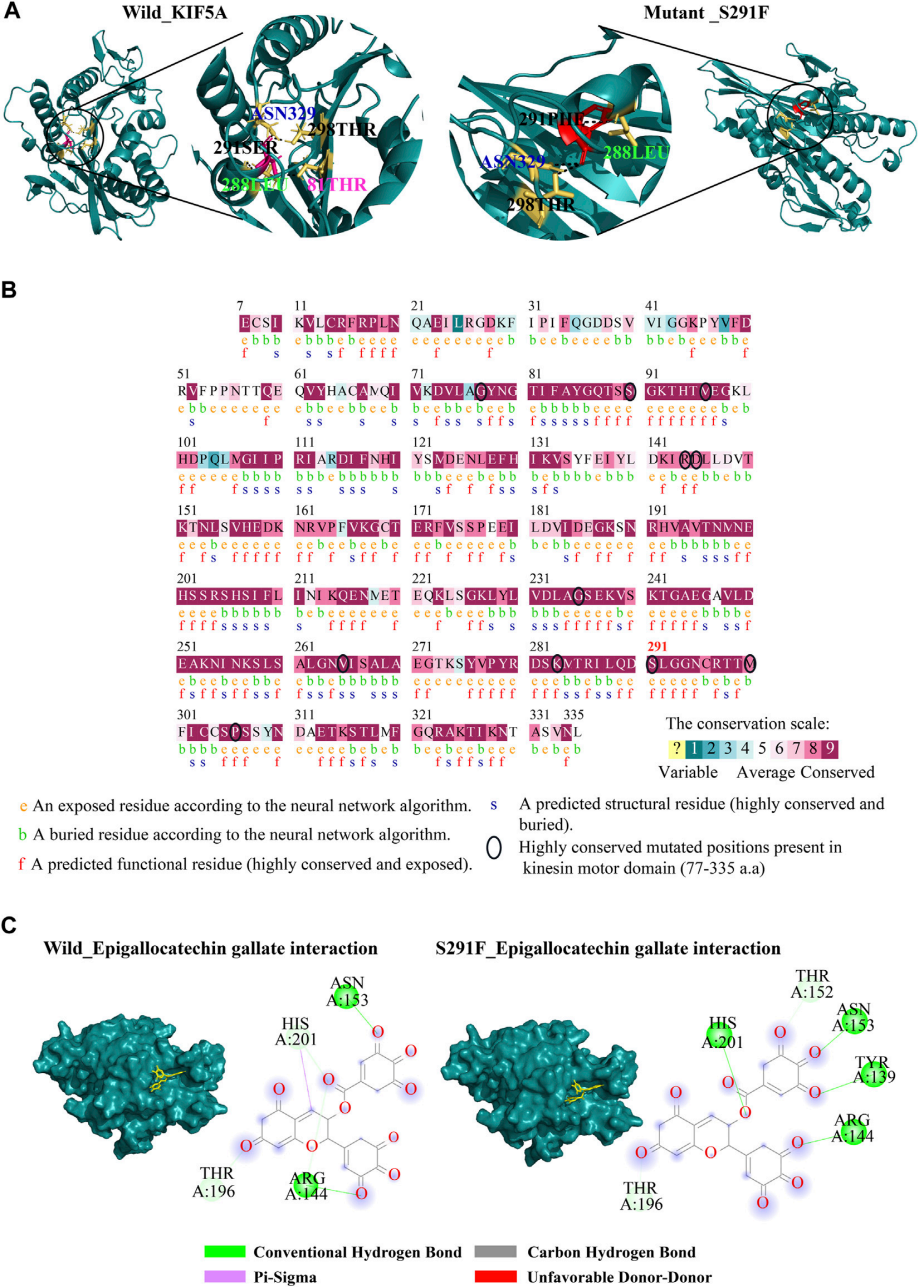
Structure analysis of wild-types and mutants. **(A)** Showing hydrogen bonds formed by wild-type and S291F mutant residues in the kinesin motor domain. The wild-type residue Ser291 (pink stick model) formed four H-bonds with THR81, LEU288, THR298, and ASN329, and the mutant residue PHE291 (red stick model) showed three H-bonds (LEU288, THR298, and ASN329). **(B)** Highlighting of evolutionary conservation analysis of the positions of mutant residues; the amino acid position is shown in the kinesin motor domain shown in the black circle. **(C)** Representation of the ligand interaction of epigallocatechin gallate phytocompounds with the kinesin motor domain of wild-type *KIF5A* and mutant S291F. Epigallocatechin gallate showed four H-bonds with the amino acid residues (TYR139, ARG144, ASN153, and HIS201) as compared to the wild-type ones.

The 3D structure for the *KIF5A* protein was downloaded using AlphaFold (ID: AF-Q12840) from the UniProt database. The downloaded model is considered a good model for structure analysis and contains a complete structure of 1–1,032 amino acid residues (Figure 2A). To validate the structure, the PDBsum and PROCHECK servers were employed, with the PROCHECK program generating a Ramachandran plot (RC plot) to assess the stereochemical characteristics of the protein structure and provide detailed residue-by-residue and overall geometry analysis. The predicted model’s reliability was confirmed, with 88.4% of residues located in the most favored region, 6.5% in the allowed region, and 2.6% in the generously allowed region, while only 2.5% of residues were situated in the disallowed region (Figure 2B). The structure stability analysis result by FoldX predicted that out of a total of 43 nsSNPs, S291F showed the highest structure-destabilizing behavior in terms of free energy (Figure 2C). The *KIF5A* ^S291F^ mutation was also presented in the kinesin motor domain (7–335 amino acids) of the *KIF5A* protein predicted by the InterPro tool (Figure 2D). To validate the structure stability differences, we checked the number of hydrogen bonds between wild *KIF5A* and variant *KIF5A* ^S291F^. The wild-type *KIF5A* ^SER291^ residue formed three hydrogen bonds with residues 81THR, 288LEU, 298THR, and ASN329, but in the case of mutant PHE291, only three hydrogen bonds were formed with 288LEU, 298THR, and ASN329, as shown in Figure 3A.

### Evolutionary conservation profile

The evolutionary conservation profile was identified for the amino acid position of highly destabilizing SNPs presented in the domain. The conservation scores calculated by this server range from 1 to 9 and discriminate between highly variable and highly conserved positions, respectively. The results included eight positions (77, 90, 96, 235, 265, 283, 291, and 306) with a score of 9 indicating that the pathogenic nsSNPs affect evolutionary conserved positions in the *KIF5A* protein as compared to the three positions (144, 145, and 300) with a score of 8 (Figure 3B).

### Bioactive compounds and molecular docking

We fetched the 24 plant-derived bioactive compounds against ALS. These plant-derived bioactive compounds associated with excitatory amino acid toxicity (β-asarone, Huperzine A, catalpol, selaginellin, ferulic acid, and cryptotanshinone), neuroinflammation (celastrol, resveratrol, curcumin, isorhynchophylline, obovatol, paeonol, and wogonin), calcium cytotoxicity (paeoniflorin, ligustrazine, gastrodin, and muscone), and oxidative stress (madecassoside, ampelopsin, epigallocatechin gallate (EGCG), picroside II, morroniside, astragaloside IV, and diallyl trisulfide) (Table 2). The docking analysis results suggested that epigallocatechin gallate showed the highest binding affinity (−9.0 kcal/mol) toward the KIF5A^S291F^ mutant present in the kinesin motor domain. We observed a comparative binding study with the wild-type *KIF5A* domain, in which EGCG showed less binding affinity (−8.4 kcal/mol), forming two hydrogen bonds with ARG144 and ASN153 position residues and four H-bonds with ARG144, ASN153, TYR139, and HIS201 residues in case of KIF5A^S291F^. Formation of a greater number of H-bonds between EGCG and KIF5A^S291F^ provides more binding strength to the complex and makes it more stable. The interacting residues between EGCG with the wild-type and the KIF5A^S291F^ kinesin motor domain are shown in Figure 3C. The binding affinity of other bioactive compounds is shown in Table 2.

**TABLE 2:** List of phytocompounds used against the KIF5A^S291F^ mutant and their binding affinity.

Phytocompound against ALS	Extracted from	ID	Binding affinity with the mutant (kcal/mol)
Epigallocatechin gallate	Green tea	ZINC000003870412	−9
Celastrol	*Tripterygium wilfordii*	ZINC000019795938	−8.4
Paeoniflorin	*Paeoniae radix*	ZINC000101324555	−8.3
Astragaloside IV	*Radix astragali*	PubChem:13943297	−8.2
Ampelopsin	*Ampelopsis grossedentata*	ZINC000013358407	−7.9
Madecassoside	*Centella asiatica*	ZINC000252448790	−7.8
Selaginellin	*Saussurea pulvinata*	ZINC000049055574	−7.5
Cryptotanshinone	*Salvia miltiorrhiza*	ZINC000002109876	−7.4
Picroside-II	*Picrorhiza kurroa*	ZINC000008382348	−7.2
Huperzine-A	*Huperzia serrata*	ZINC000100004253	−6.7
Isorhynchophylline	*Uncaria rhynchophylla*	ZINC000012412601	−6.5
Morroniside	*Cornus officinalis*	ZINC000056874303	−6.4
Resveratrol	*Veratrum nigrum*	ZINC000000006787	−6.4
Wogonin	*Scutellaria root*	ZINC000000899093	−6.3
Catalpol	*Rehmannia glutinosa*	ZINC000008234298	−6.2
Muscone	Natural muskies	ZINC000004269341	−6.1
Curcumin	*Curcuma longa*	ZINC000100067274	−5.6
Obovatol	*Magnolia officinalis*	ZINC000043059310	−5.3
Gastrodin	*Gastrodia elata*	ZINC000003881790	−5.3
Ferulic acid	Angelica and Szechwan	ZINC000000058258	−4.8
β-Asarone	*Acorus tatarinowii*	PubChem:5281758	−4.7
Paeonol	*Paeonia suffruticosa*	ZINC000000001906	−4.5
Ligustrazine	*Rhizoma chuanxiong*	ZINC000000004042	−4.3
Diallyl trisulfide	*Liliaceae allium*	PubChem:223443825	−3.2

### *KIF5A*–PPIN and associated functional modules

**FIGURE 4:**
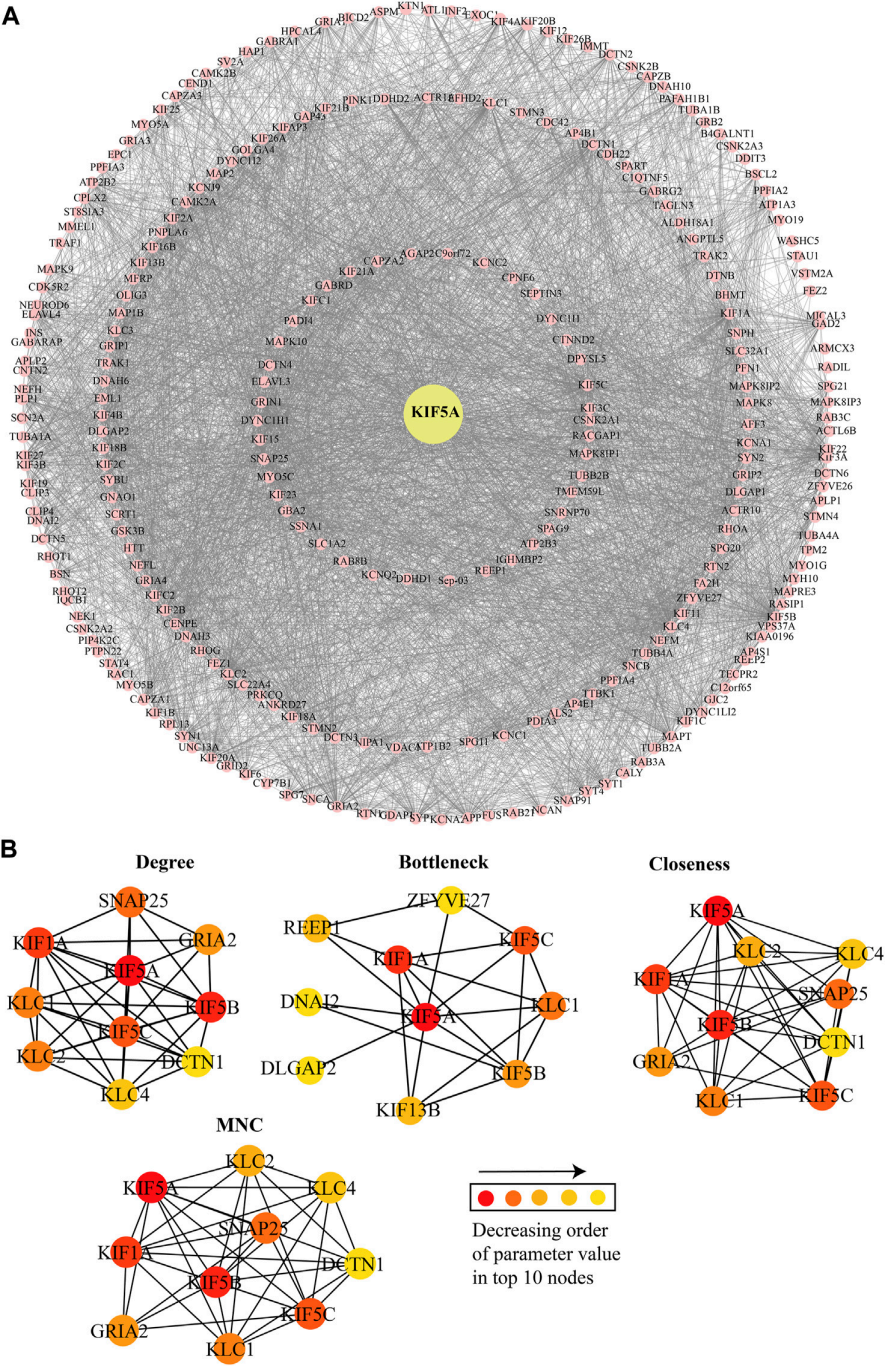
KIF5A protein–protein interaction network and its significant KIFs. **(A)** Protein–protein interaction (PPI) network of *KIF5A* (yellow-filled circle) with its associated proteins (pink-filled circle). *KIF5A*–PPIN consisted a total of 272 nodes and 4,353 edges. **(B)** Representation of top 10 nodes observed in five different network parameters; the color of the nodes in decreasing order of the value of each parameter.

**FIGURE 5:**
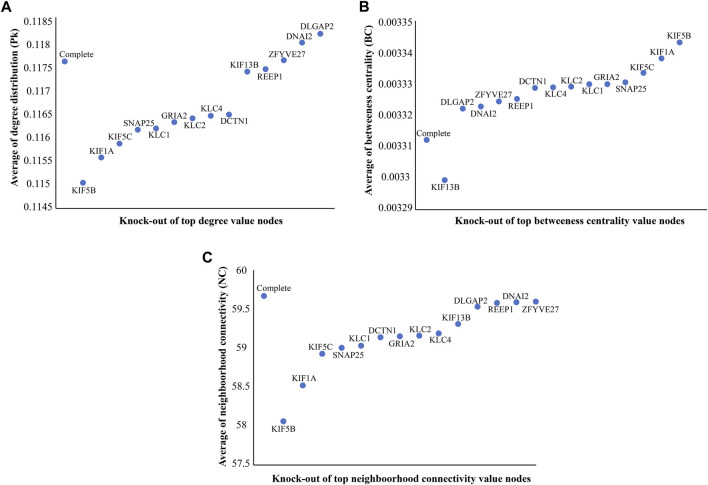
Knock-out study to identify significant nodes. **(A)** Differences of average degree distribution (Pk) values of top value degree nodes with the complete network. **(B)** Differences of average BC values of top (BC) value nodes with the complete network. **(C)** Differences of average neighborhood connectivity (NC) values of top (NC) value nodes with the complete network. We found KIFs (KIF1A, -5B, and -5C) allowed significant changes in Pk, BC, and NC, which could enhance local and global signal propagation throughout the network as compared to other nodes.

In the constructed network, we considered the directly interacting proteins of *KIF5A* and within interactions between them. The PPIN of *KIF5A* was visualized using the Cytoscape tool; the PPIN consisted of 272 nodes and 4,353 edges (Figure 4A). Topological properties suggested that KIF5A–PPIN followed a scale-free hierarchical behavior means having clusters of nodes/module organization that could be controlled by the *KIF5A* protein. We found majorly KIF motor protein associations with each other in the top 10 nodes of parameters, such as degree (*KIF5A*, *KIF5B*, *KIF5C*, and *KIF1A*), bottleneck (*KIF5A*, *KIF5B*, *KIF5C*, *KIF1A*, and *KIF13B*), closeness (*KIF5A*, *KIF5B*, *KIF5C*, and *KIF1A*), MNC (*KIF5A*, *KIF5B*, *KIF5C*, and *KIF1A*), and MNC (*KIF5A*, *KIF1A*, *KIF5B*, and *KIF5C*), parameters calculated by the CytoHubba plugin in Cytoscape (Figure 4B). We found that three KIFs (KIF1A, -5B, and -5C) were the significant proteins common in all four parameters. In the knock-out experiment, while re-examining the network’s topological property change, we found a significant magnitude of changes in network metrics in particular, such as Pk, BC, and NC (Figures 5A–C), respectively. In the local ranking method (Pk and NC) and in the global ranking method (BC), three KIFs (KIF1A, -5B, and -5C) showed a significant change in properties. The significant change in BC could enhance local and global signal propagation throughout the network (Figure 5B). The degree distribution and neighborhood connectivity showed KIFs’ (KIF1A, -5B, and -5C) control over the regulation of PPIN, whereas the removal of other nodes showed a similar pattern of regulation (Figures 5A, C).

**FIGURE 6:**
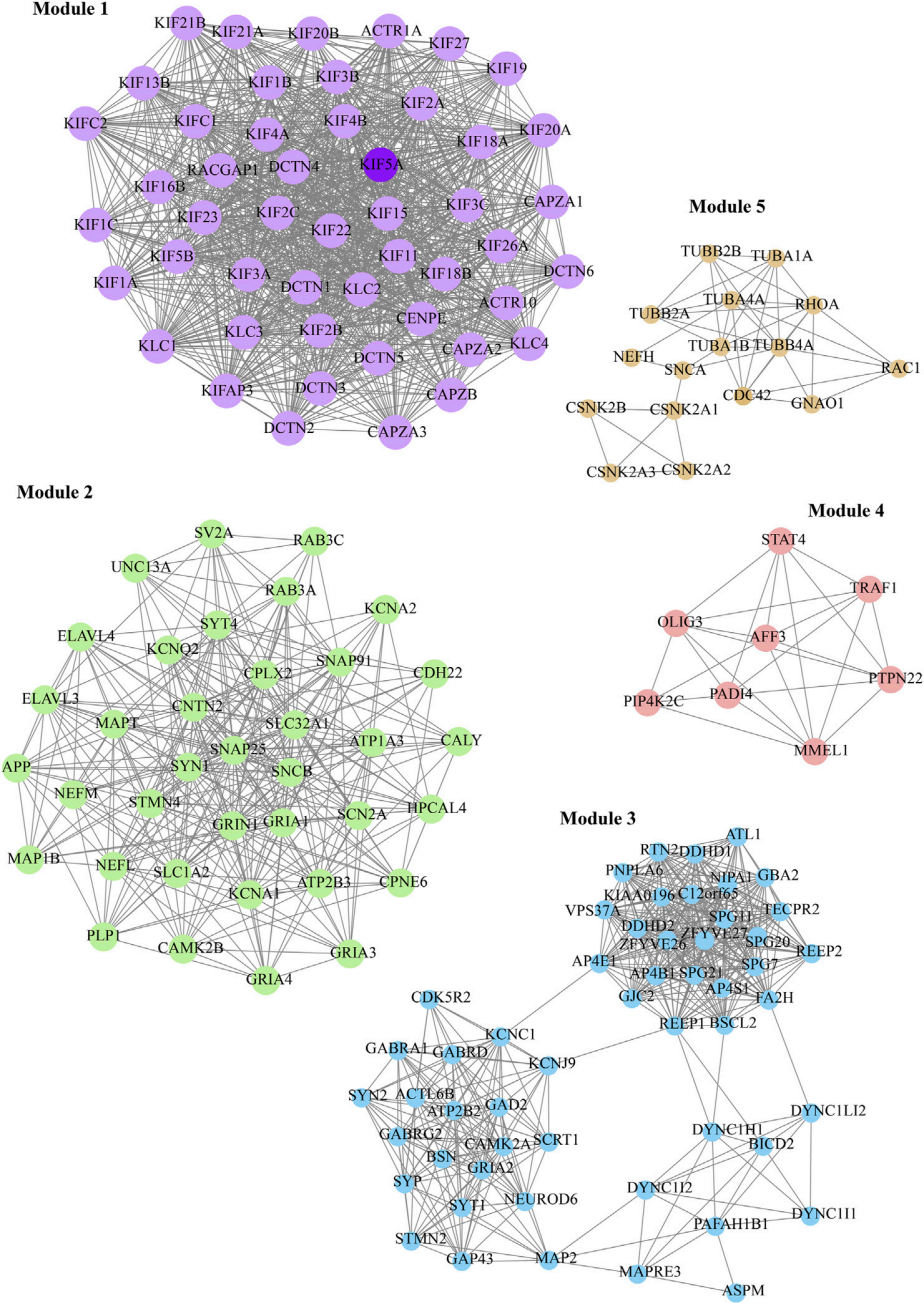
Representation of the top five functional modules presented in *KIF5A*–PPIN, shown in different colors. The *KIF5A* protein (dark purple color) is presented in the module.

**FIGURE 7:**
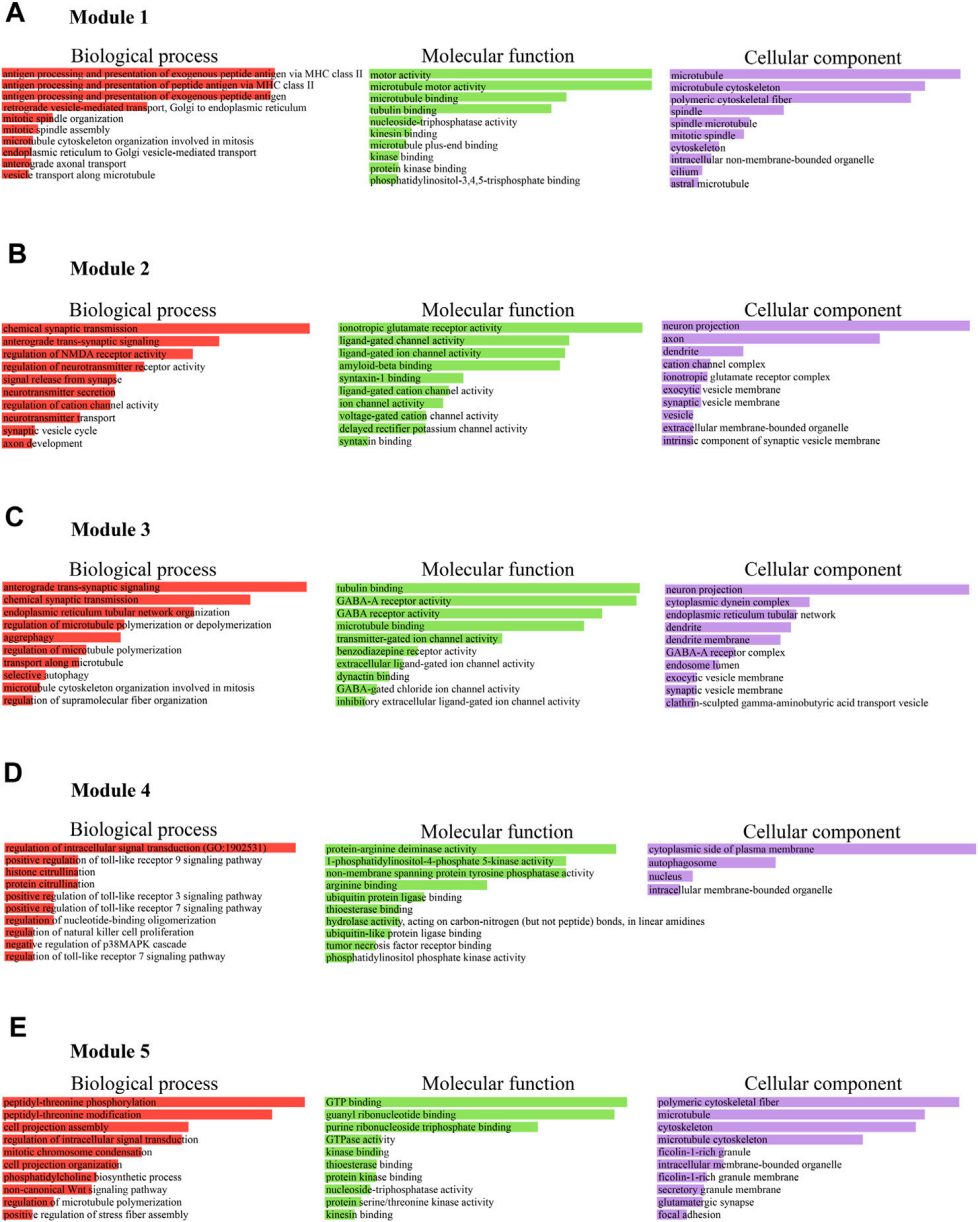
**(A)** Module 1, proteins were found to be mostly in microtubules and cytoskeletal involved in antigen processing, microtubule and motor neuron activities. **(B)** Module 2, was enriched in ligand-gated channel activity, glutamate receptor activity, and anterograde trans-synaptic and chemical synapse transmission in neuron. **(C)** Module 3 was associated to neuronic cells and is involved in tubulin binding and GABA-A receptor activity, anterograde trans-synaptic and chemical synapse transmission. **(D)** Module 4, protein was associated with protein-arginine deiminase, regulation of intracellular signal transduction, and positive regulation of Toll-like receptor 9 signaling pathway and presented in the cytoplasmic side of the plasma membrane and autophagosomes. **(E)** Module 5 proteins, were mainly present in microtubules and were cytoskeletal, performing molecular functions such as GTP binding and GTPase activity peptidyl-threonine phosphorylation.

Furthermore, we considered the top five M-score modules in which the nodes were highly clustered (Figure 6). Module 1, 2, 3, 4, and 5 consisted of 49, 37, 53, 7, and 16 nodes with the corresponding M-scores of 41.70, 18, 17.346, 6.85, and 6, respectively. The identified top five modules may regulate the network to prevent dissortivity and simultaneously maintain the flow of information within the ALS network. We performed functional enrichment analysis of the selected five modules. Module-1 exhibited enrichment in proteins possessing microtubule motor activity and their association with biological processes related to the processing and presentation of exogenous peptide antigens via MHC class II, as well as mitotic spindle organization. These proteins were found to be mostly in microtubules and were cytoskeletal (Figure 7A). Module 2 was enriched in ligand-gated channel activity, glutamate receptor activity, and anterograde trans-synaptic and chemical synapse transmission. These proteins were found mainly in the neuron projection, axon, and dendrites (Figure 7B). Module 3 was associated to neuronic cells and is involved in tubulin binding and GABA-A receptor activity. These proteins were also involved in anterograde trans-synaptic and chemical synapse transmission (Figure 7C). Module-4 protein was associated with protein-arginine deiminase, regulation of intracellular signal transduction, and positive regulation of Toll-like receptor 9 signaling pathway and are mainly found in the cytoplasmic side of the plasma membrane and autophagosomes (Figure 7D). Module-5 proteins were mainly present in microtubules and were cytoskeletal, performing molecular functions such as GTP binding and GTPase activity peptidyl-threonine phosphorylation (Figure 7E).

### Regulatory network of *KIF5A*

**FIGURE 8:**
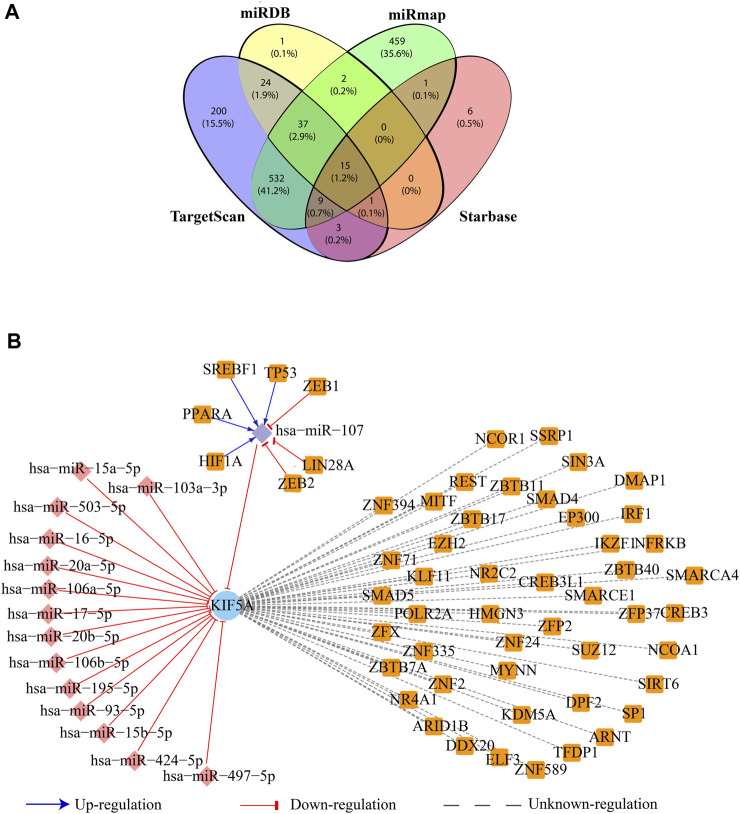
Regulatory network of the *KIF5A* protein. **(A)** Venn diagram representing the number of common miRNA targets of KIF5A in four databases. **(B)** Regulatory network of KIF5A with their associated miRNAs (diamond: pink) and transcription factors (square: orange); the mode of upregulation is shown with blue arrows and downregulation in a red T-shape.

The miRNA-target prediction tools, miRDB v6.0, TargetScan v8.0, starBase v2, and miRmap, predicted 80, 821, 1,055, and 35 miRNA targets of *KIF5A*, respectively. All these four programs depicted 15 common miRNA (hsa-miR-107, hsa-miR-16-5p, hsa-miR-15a-5p, hsa-miR-17-5p, hsa-miR-20a-5p, hsa-miR-195-5p, hsa-miR-497-5p, hsa-miR-103a-3p, hsa-miR-15b-5p, hsa-miR-93-5p, hsa-miR-106b-5p, hsa-miR-20b-5p, hsa-miR-106a-5p, hsa-miR-503-5p, and hsa-miR-424-5p) targets of *KIF5A* (Figure 8A). The TF-target prediction tool, NetworkAnalyst tool, predicted 47 TFs regulating *KIF5A*. The common 15 miRNA–TF relationships predicted by the TransmiR database showed that hsa-miR-107 was upregulated by four TFs (HIF1A, PPARA, SREBF1, and TP53) and downregulated by three TFs (ZEB1, LIN28, and ZEB2) (Figure 8B).

## Discussion

SNPs, or single-nucleotide polymorphisms, are a pivotal genetic factor contributing to ALS. Specifically, non-synonymous SNPs, which lead to alterations in a single-amino acid residue, play a significant role in ALS and various other neurological disorders. In this study, we employed both structure- and sequence-based computational tools to identify mutations impacting the structure and function of the *KIF5A* protein, potentially playing a critical role in disease onset. The mutation on the 291st position from the serine to phenylalanine residue was found to be highly destabilizing for the protein structure. The 291st residue exhibited a notably more evolutionary conservation score (Figure 3C), indicating that any mutation occurring at this specific position would likely disrupt the protein’s normal functionality. From this, interestingly, we found the S291F variant was present in a large globular N-terminal domain (kinesin motor domain) (Figure 2D) which may affect the associated protein functions and related pathways in motor neurons. A systems biology approach can be applied to anticipate regulators (such as genes, transcription factors, and microRNAs) relevant to ALS and assess their resilience in preserving self-organized behavior. This approach also helps uncover the complexities of signaling within fundamental cellular processes like cell survival and death, offering insights for developing strategies to understand motor neuron cell function. *KIF5A* functions as a motor protein responsible for the microtubule-based transport of intracellular organelles, RNA, and proteins (Hirokawa et al., 2010). KIF5A aids in RNA and its binding protein trafficking within the dendritic and axonal area. *KIF5A* plays a crucial role in facilitating the anterograde transport of mitochondria, aided by adapter proteins like TRAK1/2 and GABARAP. This function is vital for sustaining the capability and proper functions of neurons. Aberrant regulation of mitochondrial transport is linked to multiple neurodegenerative disorders (Sheng and Cai, 2012; Schwarz, 2013). ALS has recently been linked to a mutation in the cargo binding C-end domain region of KIF5A that affects exon-27 splicing (Nicolas et al., 2018). *KIF5A* plays a pivotal role in axonal transport, shedding light on the possibility that mutations in *KIF5A* can disrupt this process, thus contributing to the development of motor neuron degeneration (Hirokawa et al., 2010). A mutation in *KIF5A* could result in the accumulation of neurofilaments and hinder the transport of mitochondria, potentially serving as a hallmark of neurodegenerative diseases (Al-Chalabi et al., 1999; Palomo and Manfredi, 2015; Guo et al., 2017; Smith et al., 2017). Research conducted in zebrafish has revealed that a loss-of-function mutation in the C-terminal region leads to truncation and disrupts the proper axonal localization of mitochondria (Campbell et al., 2014). Within neuronal cells, reduced *KIF5A* expression and cargo binding lead to the buildup of amyloid precursor proteins and phosphorylated neurofilaments, serving as a distinctive hallmark of neurodegeneration (Hares et al., 2017).

Currently, only two FDA-approved drugs, namely, riluzole and edaravone, are available on the market for the treatment of ALS that provide limited benefits to the patients (Sadr et al., 2021). However, many studies have attempted to create an effective therapeutic target for ALS previously. Most drugs have completed preclinical animal trials, but human clinical trials have had underwhelming results. On the other hand, herbal remedies serve as an additional and alternate kind of treatment for ALS (Mohi-ud-din et al., 2021). The phytochemicals from plant sources, such as flavonoids, alkaloids, terpenes, and saponins, may still have the potential benefits that researchers are seeking because of their distinctive chemical diversity (Zeng et al., 2021; Shareena and Kumar, 2022 Solanki et al., 2015). Therefore, these natural substances are still unexplored as new therapeutic molecules for the treatment of ALS. For ALS, the phytochemicals isolated from medicinal herbs function in a variety of mechanisms, including as an antioxidant, an anti-inflammatory, and an anti-apoptotic agent. The demand for utilizing natural products in the treatment of ALS has grown due to their superior safety and effectiveness in comparison to conventional drugs, and their contribution has a viable alternative for treatment. In this study, the docking analysis showed EGCG bound with mutant S291F with the highest affinity (Table 2). The EGCG phytocompound present in green tea is a radical scavenger that mediates the antioxidant action in a number of neurodegenerative disorders, including ALS. An *in vivo* study reported that in the SOD1^G93A^ transgenic mice model, epigallocatechin gallate significantly delays the disease onset by 1.4 weeks and prolongs the survival time by 1.8 weeks (Mandel et al., 2004). Epigallocatechin gallate prevented the oxidation stress-induced apoptosis of motor neurons by upregulating survival signals, including phosphatidylinositol 3-kinase, phospho-Akt, and phospho–glycogen synthase kinase-3 (pGSK-3) (Koh et al., 2004). EGCG had an inhibitory effect on GSK-3 activity, one of the important pathogenic mechanisms of an *in vitro* model of familial ALS (Koh et al., 2005). The presence of ROS and RNS will intensify protein misfolding. Experimental research has emphasized the potential of EGCG to function as an antioxidant, hindering the conversion of nitrate and peroxynitrite into NO. This action is believed to reduce ischemic neuronal harm and safeguard nerve cells (Nagai et al., 2002). Here, our finding suggested that out of the 24 phytocompounds, EGCG binding with *KIF5A* ^S291F^ could be a potential neuroprotective drug target for ALS disease.

The PPIN analysis revealed that *KIF5A* may be a crucial protein for ALS pathogenesis, controlling the function of the associated functional modules involved majorly in microtubule motor activities in the neuron. The network analysis approach resulted in a number of KIFs (*KIF5A*, *KIF5B*, *KIF5C*, *KIF1A*, *KIF13B*, *KIF3C*, *KIF11*, *KIF18A*, and *KIF22*) associated with *KIF5A* and was observed in the top 10 nodes for parameters such as degree, bottleneck, closeness, and MNC (Figure 4B). These KIFs are involved in excitatory synaptic transmissions, like *KIF13B*, and *KIF11* regulates synaptic transmission through post-synaptic and/or pre-synaptic mechanisms (Swar et al., 2018). Three *KIF5A*, *KIF5B*, and *KIF13B* transporting cargos, two in microtubule rearrangements (*KIF3C* and *KIF11*), and *KIF11*, a mitotic KIF, are involved in spindle movement. Computational knockout experiment suggested that KIFs (KIF1A, -5B, and -5C) show a significant amount of changes in network properties, particularly in Pk, BC, and NC (Figure 5). These KIFs could enhance local and global signal propagation throughout the network (Figures 5A–C). One study reported that *KIF5A* conditional knockout mice significantly reduced the amplitude of mIPSCs (Nakajima et al., 2012). Furthermore, the regulatory network analysis showed that *KIF5A* gene expression can be regulated by both transcriptional and post-transcriptional factors (miRNAs). We found *KIF5A* appears to be predominantly directly or indirectly co-regulated by a number of TFs and miRNAs. Our regulatory network analysis has revealed that the expression of the *KIF5A* gene can be regulated at both the transcriptional and post-transcriptional levels, particularly by miRNAs. Our findings indicate that *KIF5A* is predominantly regulated, either directly or indirectly, by a range of transcription factors and miRNAs. Our analysis suggests that abnormal *KIF5A* gene expression due to mutations can be directly inhibited by 15 miRNAs (Figure 8B) and indirectly regulated by seven TFs (HIF1A, PPARA, SREBF1, TP53, ZEB1, LIN28, and ZEB2). Specifically, miR-107 directly inhibits the abnormal KIF5A expression, while TFs associated with both the upregulation (HIF1A, PPARA, SREBF1, and TP53) and downregulation (ZEB1, LIN28, and ZEB2) of miR-107 contribute to the indirect control of *KIF5A* expression.

## Conclusion

Understanding the biological effects and mechanisms of action of the newly found phytochemicals can be accomplished by testing compounds using *in vitro* KIF5A^S291F^ knockout model systems. Further validation of *in vitro* and *in vivo* conditions would help us in the identification of key immunological pathways and processes involved in the progression of ALS. In conclusion, the validation of the identified compound EGCG through the appropriate knockout model system (KIF5A^S291F^) may hold great promise for advancing our understanding of its potential therapeutic applications. As we move forward, it is essential to meticulously select model systems that closely mimic the specific conditions or disease. For instance, in the context of studying EGCG’s anti-cancer effects, the use of cancer cell lines with targeted gene knockouts related to cancer pathways can provide valuable insights (Almatrood et al., 2020). EGCG could serve as a “targeted ALS therapy,” which refers to a new class of medications developed to particularly block *KIF5A* and its mutations, thought to be essential for ALS development or progression. The generation of transgenic knock-out model organisms (*KIF5A* ^S291F^) would provide an understanding, development, and pathogenesis of ALS, as well as for the evaluation of possible risk factors, preventive agents, and treatments. In future, experimental approaches may encompass a comprehensive strategy that includes control groups with wild-type and knock-out models, specifically targeting the *KIF5A* gene. Utilizing the computational analysis of gene knock-outs, we observed substantial alterations in network metrics, especially in degree distribution and betweenness centrality. This significant change in degree distribution and betweenness centrality could enhance local and global signal propagation throughout the network, followed mutually by KIF1A, -5B, and -5C. The association between KIF proteins and knock-out studies of such proteins would provide a basis for the identification of biomarkers that could be used as therapeutic interventions. Finally, this study may help us predict the key molecules (phytocompounds, miRNAs, and TFs), and the molecular interactions between them may provide an opportunity for understanding ALS pathology and therapeutic targets.

## Data Availability

The original contributions presented in the study are included in the article/Supplementary Material; further inquiries can be directed to the corresponding authors.
